# The NDUFV2 gene silencing inhibits the proliferation of two drug-resistant cancer cell lines

**DOI:** 10.1186/s43141-022-00343-2

**Published:** 2022-04-26

**Authors:** Lingling Liu, Xunan Wang, Yue Li, Chengyao Ma, Yeye Shi, Xiang Li, Jianwei Chen

**Affiliations:** School of Pharmacy, Nanjing University of Chinese Medicine, Nanjing, 210023 Jiangsu Province China

**Keywords:** NDUFV2, Gene silencing, Drug resistance, Cancer, Western blot, qRT-PCR, Transfection, New target

## Abstract

**Background:**

Cancer is a group of diseases involving abnormal cell growth with the potential to invade or spread to other parts of the body. It is unlikely that there will ever be a single cure for cancer, but the development of molecular biology and cell biology has brought new options for cancer treatment. Our research group found in the preliminary experiments that AAs exhibited significant anti-tumor activity. Studies also showed that AAs exhibited varying degrees of downregulation effects on the expression of the NDUFV2 gene in the MCF-7/ADR and SMMC-7721/ADR cell lines. However, there is no relevant report on the role of this gene expression during the growth process of drug-resistant tumor cells. To address possible objections, this paper aims to investigate the effect of NDUFV2 gene silencing on the proliferation of the MCF-7/ADR and SMMC-7721/ADR cell lines.

**Results:**

The interfering plasmids pPLK/GFP+Puro-NDUFV2 shRNA-3 and shRNA-2 inhibited the NDUFV2 gene and protein expression most significantly in MCF-7/ADR and SMMC-7721/ADR cells, respectively. NDUFV2 gene silencing could effectively inhibit the proliferation of both cell lines. The inhibition rates for MCF-7/ADR were 67.31%, 73.02%, and 69.76% at 24 h, 48 h, and 72 h, while that for SMMC-7721/ADR were 68.89%, 71.97%, and 74.40% at 24 h, 48 h, and 72 h, respectively. The inhibition rate of SMMC-7721/ADR cell proliferation was positively correlated with time.

**Conclusions:**

Interference with the NDUFV2 gene may significantly inhibit the proliferation of MCF-7/ADR and SMMC-7721/ADR cells. This study is the pioneer to investigate that the NDUFV2 gene has been associated with the activity of inhibiting tumor cell proliferation, suggesting that the NDUFV2 gene may become a potential target for the study of tumor genesis and the development of antineoplastic drugs.

## Background

Drug resistance continues to be the principal limiting factor to achieving cures in patients with cancer. Surgery, radiotherapy, and polychemotherapy were administered in combination from the rulebook of antimicrobial therapy [1] and clearly not enough to cure many tumor types. In some cases, the effects of therapies (mostly chemotherapy) can be profound and equivalent to inducing a state of genomic instability, which prompts us to weigh carefully the potential unintended consequences of administering chemotherapy or radio therapy [2, 3]. Also, despite a growing number of successes in efforts to target oncogenic driver mutations, some of the most formidable oncogenes and tumor suppressor genes remain undruggable and are still under research, such as MYC [4], RAS [5], and TP53 [6]. Therefore, combining an assessment of the physical properties of the tumor together with a deep analysis of tumor drivers and druggability, dependencies and vulnerabilities will be a challenging but the formative approach to fighting the resistance of cancer to therapy [7].

Natural products have provided a rich source of chemical structures for the development of anti-cancer treatments. The AAs are secondary metabolites produced by the Annonaceae family [8]. They are characterized by a long aliphatic chain containing 35–37 carbon atoms with a methyl-substituted or rearranged α, β-unsaturated γ-lactone ring at the terminal, and 0–3 tetrahydrofuran (THF) rings [9]. In modern pharmacology, AAs are rendered as important antitumor agents for their outstanding performance in the process that is closely related to tumor metabolism, cell death, apoptosis, and autophagy [8]. So far, AAs are found to exert their cytotoxic activity against neoplasm through five ways where the most prominent activity is the inhibition of the Mitochondrial Complex І in the respiratory chain of tumor cells [10]. Besides, they can also induce and enhance apoptosis of tumor cells by upregulating apoptosis effectors or proapoptotic genes [11, 12] and downregulating antiapoptotic genes [13], inhibit tumor cell proliferation by cell cycle arrest [12] and targeting the aerobic glycolysis process [14], and suppress autophagy [15]—a process which can provide cancer cells with high metabolic requirements for cell proliferation by recycling macromolecules, non-functional organelles, and proteins [16]. Last but not least, AAs are demonstrated to possess downregulating effect on the MDR1 and MRP1 genes, thus reversing drug resistance to human hepatocellular carcinoma [17]. Thus, AAs are not only promising anti-neoplastic agents, but also new possible therapeutic molecules against drug-resistant cancer cells.

Our research group found in the preliminary experiments that AAs possess varying degrees of inhibition effect on the proliferation of drug-resistant cancer cell lines, such as A549/taxol [18] and MCF-7/ADR [9]. In addition, AAs are also found to downregulate the expression of the NDUFV2 gene in MCF-7/ADR [19] and SMMC-7721/ADR [20] cell lines. Factors affecting the downregulation of the NDUFV2 gene by AAs include the number of carbons between the tetrahydrofuran ring and the lactone ring, the number of substituted hydroxyl groups, the tetrahydrofuran ring conformation, and the number of carbons between the tetrahydrofuran ring and the long-chain alkyl terminus [20].

It has been shown that the expression of the NDUFV2 gene in MCF-7/ADR and SMMC-7721/ADR cells is differentially downregulated by AAs, but the role of this gene expression in the growth of drug-resistant tumor cells has not been reported. To disprove the constitutive relationship, three NDUFV2 interfering plasmids and one negative control empty plasmid were transiently transfected with Lipofectamine^TM^ 2000 in MCF-7/ADR and SMMC-7721/ADR cells. The mRNA expression of the NDUFV2 gene was detected by qRT-PCR, and the protein expression of NDUFV2 was detected by Western blot in each group of tumor cells after transfection. The plasmids that were screened out with the best NDUFV2 gene silencing activity were then used to determine their inhibitory effects on the proliferation of the two drug-resistant tumor cells by MTT colorimetric assay.

In summary, the aim of this study was to construct a relationship between NDUFV2 gene downregulation and drug-resistant tumor cell proliferation, thereby demonstrating that AAs can inhibit the proliferation of drug-resistant tumor cells by downregulating NDUFV2 gene expression.

## Methods

### Reagents

ReverTra Ace^TM^ qPCR RT Master Mix with gDNA Remover (FSQ-301, Toyobo Co., Ltd.), SYBR® Green Realtime PCR Master Mix (QPK-201, Toyobo Co., Ltd.), 6×loading buffer (Nanjing KeyGen Biotech Co., Ltd.), Tris-base (Sigma-Aldrich LLC.), Glycine, Trypsin, TEMED, Tween 2 (Biosharp Biotechnology Co., Ltd.), NaCl (Sinopharm Group), PageRuler™ Prestained Protein Ladder, Lipofectamine^TM^ 2000 (Thermo Fisher Scientific Inc.), NDUFV2 prime, HMBS prime (Invitrogen by Thermo Fisher Scientific Inc.), MicroAmp® Optical Adhesive Film, MicroAmp® Optical 96-Well Reaction Plate with Barcode (Applied Biosystems by Thermo Fisher Scientific Inc.), Anti-β-Actin Antibody, NDUFV2 Rabbit PolyAb, HRP-conjugated Affinipure Goat Anti-Rabbit IgG(H+L) (Proteintech group, Inc.), DMEM media (Wisent Biotechnology (Nanjing) Co., Ltd), DEPC H2O, fetal bovine serum (FBS) (Zhejiang Tianhang Biotechnology Co., Ltd), methyl thiazolyl tetrazolium (MTT), phosphate-buffered solution (PBS), ethylene diamine tetra-acetic acid (EDTA), Penicillin-Streptomycin Solution (100X) (Beijing Solarbio Science & Technology Co., Ltd.), pPLK/GFP+Puro-NDUFV2, pPLK/GFP+Puro plasmid (Bioworld Technology, Inc.), PMSF (ST506), cell lysis buffer for Western and IP (P0013), Enhanced BCA Protein Assay Kit (P0010S) (Shanghai Beyotime Biotechnology Co., Ltd), TGX Stain-Free™ FastCast™ Acrylamide Kit, 12%, ammonium persulfate (Bio-Rad Laboratories, Inc.), Immobilon Western Chemiluminescent HRP Substrate (WBKLS0100), and PVDF membranes (Millipore Sigma). Other reagents were of analytical grade and commercially available.

### Transfection

Cells at the logarithmic growth stage were inoculated into 6-well plates with complete culture media at a density of 2×10^5^ cells/mL and 2 mL per well and incubated at 37 °C in a 5% CO_2_ incubator until the cell confluency reached 80%. Cells were divided into blank group (no treatment), negative control group (transfected with empty plasmid pPLK/GFP + Puro), and positive control group (transfected with pPLK/GFP + Puro-NDUFV2 shRNA-1, 2, and 3). Before transfection, the nutrient solution in the 6-well plates was replaced with a serum-free cell culture solution.

Transfections were performed using Lipofectamine reagent according to the instructions of the manufacturer. Briefly, 8 μg of plasmids was added to 250 μL of serum-free culture media and mixed thoroughly in a 1.5 mL centrifuge tube. Then, 8 μL of Lipofectamine^TM^ 2000 was added to 250 μL of serum-free culture media in another tube, mixed thoroughly, and stood for 5 min at room temperature. The plasmid solution was then mixed with LipofectamineTM 2000 solution and left to stand for 20 min at room temperature. Five hundred microliters of the above mixture were added to each well of the six-well plate and incubated for 6 h at 37 °C in a 5% CO_2_ incubator. When the cells were attached, the original solution was replaced with 2 mL/well of serum-containing cell culture media.

The sequences of three positive interfering plasmids pPLK/GFP + Puro-NDUFV2 were as follows:

**Table:** 

shRNA-1:5′-CCAGTTGGAAAGTATCACATT-3′ shRNA-2:5′-CCTCCAATGAGAGTATATGAA-3′ shRNA-3:5′-CCATTTGATTTCACACCAGAA-3′	

The expression level of the NDUFV2 gene in each group was detected 24 h after transfection.

### qRT-PCR assay

#### Total RNA extraction

Cells were placed on ice and washed twice with pre-cooled PBS, and the residue was aspirated. One milliliter of TRIzol reagent was added and mixed thoroughly by pipetting. Then, the homogenate was transferred into a 1.5-mL sterile centrifuge tube. Three hundred microliters of chloroform were added, shaken vigorously for 15 s, let stand on ice for 5 min, and then centrifuged at 4 °C for 15 min at 12000 r/min. The aqueous phase of the sample was aspirated and transferred to a new 1.5-mL sterile centrifuge tube. Six hundred microliters of 100% isopropanol was subsequently added, let stand on ice for 5 min, and centrifuged at 4 °C for 10 min at 12000 r/min. The supernatant was discarded. The RNA precipitate was then washed with 75% ethanol-DEPC water and centrifuged at 4 °C for 5 min at 7500 r/min. The washing solution was then discarded. The RNA precipitate was dried in a fume hood for 10 min, and 20 μL of DEPC water was used to dissolve the RNA precipitate. The total RNA sample was stored at −80 °C until use.

#### Determination of RNA concentration

RNA concentration of the above-extracted samples was determined using NanoDrop™ One (A260 / 280> 1.8).

#### cDNA synthesis

Preparation of Reaction Solution I: 440 μL of 4×DNA Master Mix and 8.8 μL of gDNA fraction were homogenously mixed, briefly vortexed and dispensed, and stored at −20 °C. Preparation of Reaction Solution II on ice: 2 μL of Reaction Solution I, 0.5 μg of the RNA template, and an appropriate amount of nuclease-free water were mixed to a final volume of 8 μL, vortexed briefly, and warmed in a 37 °C water bath for 5 min. Preparation of Reaction Solution III on ice: 8 μL of reaction solution II and 2 μL of 5×RT Master Mix II were mixed, vortexed briefly, and put in a 37 °C water bath for 15 min, and then in a 98 °C water bath for 5 min to obtain cDNA reaction solution. The cDNA reaction solution was stored at −20 °C, pending use.

#### qRT-PCR reaction

The reaction conditions are illustrated in Tables 1, 2, 3, and 4.

**Table 1:** qRT-PCR reaction system: primers used to amplify mRNA

Primer	HMBS	NDUFV2
Upstream sequence	CCCTGGAGAAGAATGAAGTGGA	TTTAGGGGCCTGTGTGAACG
Downstream sequence	TTTGGGTGAAAGACAACAGCATC	GAGAAGCGTCCACTCCTTGG

**Table 2:** qRT-PCR reaction system: real-time fluorescent quantitative PCR reaction system

Reagent	Volume (μL)	Concentration
DEPC water	0.4	-
SYBR Green dye	10	1×
Upstream primer (10 μM)	0.8	0.4 μM
Downstream primer (10 μM)	0.8	0.4 μM
cDNA template	2	-
Total volume	20	-

**Table 3:** qRT-PCR reaction system: real-time fluorescent quantitative PCR reaction conditions (standard cycle mode)

Step	Temperature (°C)	Duration (min)	Number of cycles
UDG enzyme activation	50	2	-
Pre-denaturation	95	3	-
Denaturation	95	5	-
Annealing	55	10	50
Extension	72	15	-

**Table 4:** qRT-PCR reaction system: melting curve reaction conditions

Step	Heating rate (°C/s)	Temperature (°C)	Time (s)
1	1.6	95	15
2	1.6	60	60
3	0.15	95	15

### Western blotting

Proteins were extracted from cells in an ice-cold lysis buffer (P0013: PMSF=100: 1). Twenty micrograms of protein per lane was separated by SDS-polyacrylamide gels and followed by transferring to a polyvinylidene difluoride membrane. The membrane was blocked with 5% skim milk and then incubated with the antibodies against NDUFV2. After binding of an appropriate secondary antibody coupled to horseradish peroxidase, proteins were visualized by enhanced chemiluminescence according to the instruction of the manufacturer.

### MTT assay

The viability of SMMC-7721/ADR and MCF-7/ADR cells was measured by a colorimetric methylthiazole tetrazolium (MTT) assay. Briefly, cells were incubated for 24, 48, and 72 h at 37 °C, 5% CO_2_ after transfection. Twenty microliters of MTT were added to 200 uL per well of cells and incubated for 4 h. The formazan formed was dissolved in DMSO. The optical density was measured using a microplate reader at 490 nm. The optical density of formazan formed in control (no treatment) cells was taken as 100% of viability.

### Statistical analysis

All experiments were done at least three times. All values are displayed as the mean ± S.D. of three independent determinations. The statistical analysis was performed with analysis of the GraphPad Prism 6.0 software.

## Results

### qRT-PCR assay to detect the expression level of NDUFV2 gene after plasmid transfection

**Fig. 1:**
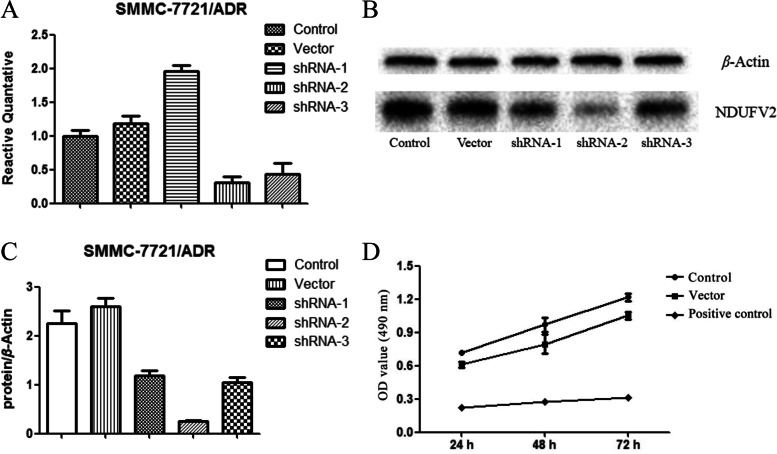
Inhibition of NDUFV2 gene silencing on the proliferation of SMMC-7721/ADR cells. **A** The relative quantitative of NDUFV2 mRNAs in each group of SMMC-7721/ADR. The expression level of the NDUFV2 gene in each group was detected 24 h after transfection. **B** The protein expressions of NDUFV2 after transfection in SMMC-7721/ADR. β-actin was used as an internal reference protein and NDUFV2 was used as the target protein. **C** The protein expressions of NDUFV2 after transfection in SMMC-7721/ADR. β-actin was used as an internal reference protein and NDUFV2 was used as the target protein. For **A**, **B**, and **C**, cells transfected with PPLK /GFP+ PURO-NDUFV2 shRNA-1, 2, and 3 were used as the positive control (shRNA-1, 2, 3), and cells transfected with empty plasmid were used as negative control (vector), and the blank control group did not do any treatment (control). **D** The growth curves of each group of SMMC-7721/ADR after transfection at different time points $\overline{x}\pm s,n=3$

To investigate the effect of positive plasmids on the NDUFV2 gene expression, we transfected cells with three different positive plasmids. qRT-PCR experiments revealed that the inhibition rates of shRNA-1, 2, and 3 plasmids on the NDUFV2 gene expression in SMMC-7721/ADR cells were −95.4%, 68.7%, and 56.5%, respectively, with shRNA-2 showing the most significant inhibition activity of the NDUFV2 gene in SMMC-7721/ADR. An 18% increase of this gene expression was observed in the negative control group compared to the blank control group (as shown in Table 5 and Fig. 1A).

**Table 5:** Inhibition of NDUFV2 gene silencing on the proliferation of SMMC-7721/ADR cells: the relative quantitative and inhibition rate of NDUFV2 in each group of SMMC-7721/ADR $\overline{x}\pm s,n=3$

Sample name	Control	Vector	shRNA-1	shRNA-2	shRNA-3
Relative quantification	1.000	1.180	1.954	0.313	0.435
SD	0.089	0.122	0.088	0.080	0.162
Inhibition rates		-18.0%	-95.4%	68.7%	56.5%

**Fig. 2:**
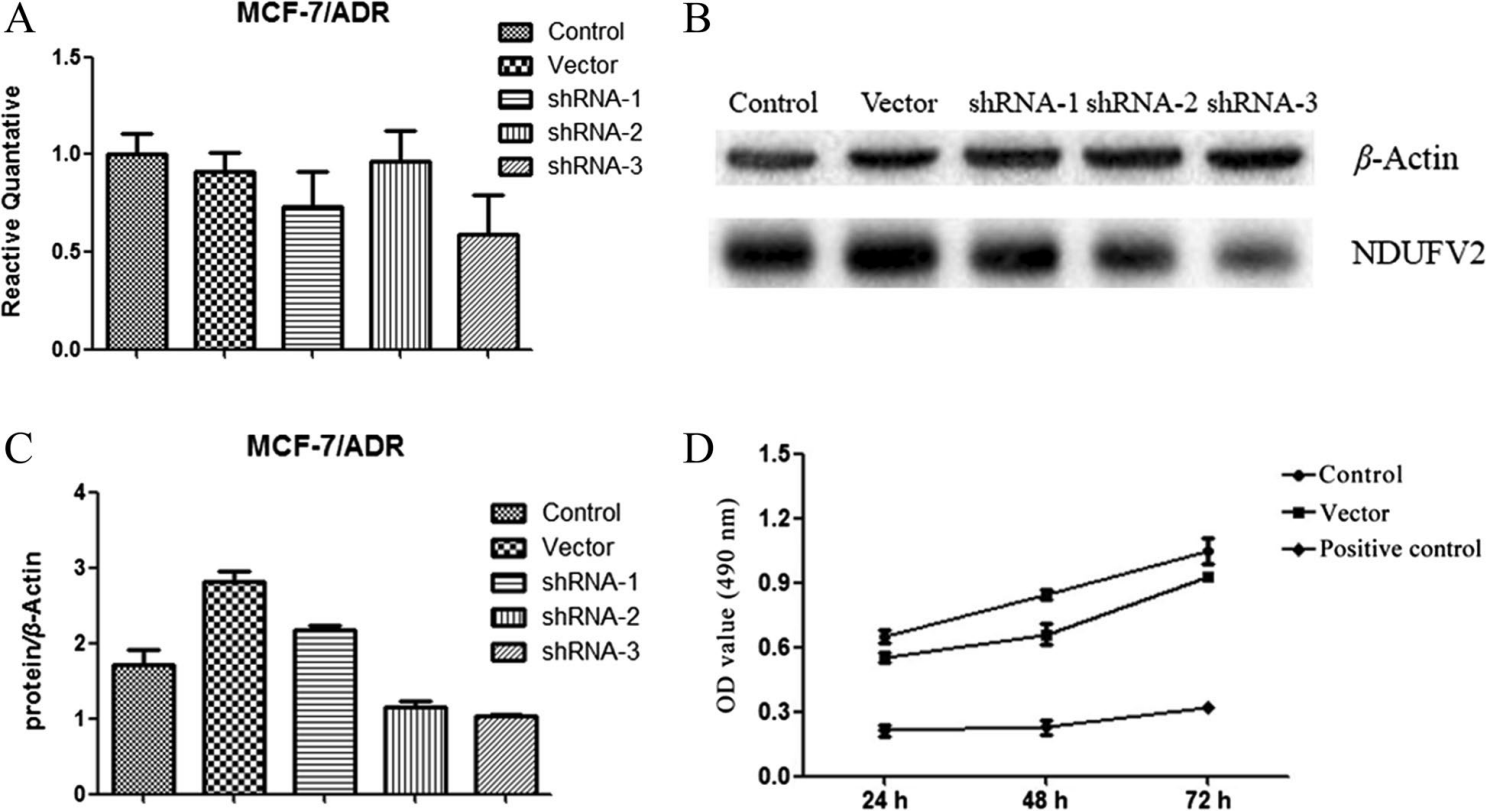
Inhibition of NDUFV2 gene silencing on the proliferation of MCF-7/ADR cells. **A** The relative quantitative rates of NDUFV2 mRNAs in each group of MCF-7/ADR after transfection at different time points $\overline{x}\pm s,n=3$. **B** The protein expressions of NDUFV2 after transfection in MCF-7/ADR cells. β-actin was used as an internal reference protein, and NDUFV2 was used as the target protein. **C** The protein expressions of NDUFV2 in MCF-7/ADR after transfection $\overline{x}\pm s,n=3$, β-actin was used as an internal reference protein and NDUFV2 was used as the target protein. For **A**, **B**, and **C**, cells transfected with PPLK /GFP+ PURO-NDUFV2 shRNA-1, 2, and 3 were used as the positive control (shRNA-1, 2, and 3), and cells transfected with empty plasmid were used as negative control (vector), and the blank control group did not do any treatment (control). **D** The growth curves of each group of MCF-7/ADR after transfection at different time points $\overline{x}\pm s,n=3$

Meanwhile, for MCF-7/ADR cells, qRT-PCR experiments revealed that the inhibition rates of three positive plasmids on the NDUFV2 gene expression were 26.5%, 3.7%, and 40.7%, respectively, with shRNA-3 showing the most significant inhibition activity. An 9.1% decrease of target gene expression was observed (as shown in Fig. 2A, Table 7).

### Western blotting to detect the protein expression of NDUFV2 after plasmid transfection

To further investigate the effect of transfected plasmids on NDUFV2, the expression levels of NDUFV2 protein in each group of cells were detected by Western blot. β-Actin was used as the internal reference protein, and NDUFV2 was the target protein.

Western blot results revealed that, compared with the blank control group, transfection of shRNA-1, 2, and 3 plasmids caused a 47.10%, 88.61%, and 53.29% downregulation of NDUFV2 protein expression in SMMC-7721/NDUFV2 cells, respectively. shRNA-2 exerted the most significant interfering effect on the expression of NDUFV2 protein in SMMC-7721/ADR. A 15% increase in protein expression was observed in the negative control group compared to the blank control group (as shown in Fig. 1B, C).

For MCF-7/ADR cells, the results demonstrated that, compared with the blank control group, transfection of shRNA-1, 2, and 3 plasmids caused a −27.47%, 32.24%, and 39.82% downregulation of NDUFV2 protein expression, respectively. shRNA-3 performed the best interfering effect on the expression of NDUFV2 protein (as shown in Fig. 2B, C).

### MTT assay to detect the effect of NDUFV2 gene silencing on cell growth

To investigate the effect of NDUFV2 gene silencing on cell viability which was detected by MTT, cells were transfected with their most positive plasmids to achieve the best inhibitory activity. shRNA-2 transfected cells are used as the positive control for SMMC-7721-ADR, while that transfected with shRNA-3 were set as the positive group for MCF-7/ADR.

The results demonstrated that the OD value of the positive control group was significantly decreased and the growth inhibition rate was 68.89%, 71.97%, and 74.40% at 24 h, 48 h, and 72 h, respectively (as shown in Table 6, Fig. 1D), indicating that NDUFV2 gene silencing could effectively inhibit the proliferation of SMMC-7721/ADR cells.

**Table 6:** Inhibition of NDUFV2 gene silencing on the proliferation of SMMC-7721/ADR cells: the growth inhibition rate of the positive control group $\overline{x}\pm s,n=3$

Time (h)	Growth inhibition rate	SD
24	68.89%	0.0053
48	71.97%	0.0014
72	74.40%	0.0063

**Table 7:** Inhibition of NDUFV2 gene silencing on the proliferation of MCF-7/ADR cells: the relative quantitative and inhibition rate of NDUFV2 in each group of MCF-7/ADR $\overline{x}\pm s,n=3$

Sample name	Control	Vector	shRNA-1	shRNA-2	shRNA-3
Relative quantification	1.000	0.909	0.735	0.963	0.593
SD	0.105	0.101	0.176	0.162	0.203
Inhibition rate		9.1%	26.5%	3.7%	40.7%

**Table 8:** Inhibition of NDUFV2 gene silencing on the proliferation of MCF-7/ADR cells: the growth inhibition rate of the positive control group $\overline{x}\pm s,n=3$

Time (h)	Growth inhibition rate	SD
24	67.31%	0.0146
48	73.02%	0.0143
72	69.76%	0.0111

For MCF-7/ADR, the results showed that the OD value of the positive control group was significantly decreased and the growth inhibition rate was 67.31%, 73.02%, and 69.76% at 24 h, 48 h, and 72 h, respectively (as shown in Table 8, Fig. 2D), indicating that NDUFV2 gene silencing could effectively inhibit the proliferation of MCF-7/ADR cells.

## Discussion

NDUFV2 gene silencing could inhibit the proliferation of drug-resistant cancer cell lines, thus indicating a potential target for cancer treatment. As our results revealed, the positive plasmid shRNA 3 performed the best inhibitory effect on the downregulation of the NDUFV2 target in the MCF-7/ADR cell line, while shRNA 2 outperformed another two plasmids in SMMC-7721/ADR cells. Our previous research discovered that like these two plasmids, AAs can also decrease the expression of the NDUFV2 gene in both cancer cell lines [19, 20]. Therefore, shRNA 3 and shRNA 2 were then selected for MTT assay to validate the cell viability of drug-resistant cancer cells MCF-7/ADR and SMMC-7721/ADR after downregulating the expression of their intrinsic NDUFV2 gene and protein. The results showed that interfering with the NDUFV2 gene significantly inhibited the proliferation of MCF-7/ADR and SMMC-7721/ADR cells, and its rate of inhibition of SMMC-7721/ADR cell proliferation was positively correlated with time; this is the first time that the NDUFV2 gene has been linked to the activity of inhibiting tumor cell proliferation, suggesting that it may become a new target for studying tumor development.

AAs are an important source of cancer treatment agents and are most famous for their inhibitory effect on the mitochondrial complex I. AAs were once observed to diminish drug-resistant human hepatocellular carcinoma by downregulating the expression of the MDR1 and MRP1 genes [17]. The results of our study specifically pointed out that AAs could also work with the NDUFV2 sub-unit of complex I to exert their anticancer function. This is also the first time to reveal that the NDUFV2-related pathways of mitochondrial complex I could have a relationship with the proliferation of tricky drug-resistant cancer cells. In addition, our research findings could direct a new target for drug-resistant cancer treatment in searching of promising curable agents of plant origin.

Resistance to therapy remains the biggest challenge in cancer today [7]. There are still some problems to be clarified in future research work in light of this topic. The first is regarding the selective therapeutic pressure of NDUFV2 gene silencing. For example, it was suggested that polymorphisms of the NDUFV2 gene may be one of the genetic risk factors for bipolar disorder [21]. In response to the downregulation of NDUFV2 gene expression, massive and widespread effects on surviving cells and non-cancer cells, and also immune responses in the host that attenuate anti-tumor responses should be further explored [7, 22]. Also, other pathways involved in the anticancer activity of AAs that co-operate or do not with NDUFV2 gene regulation need more attention. In other words, we still need to fully understand key tumor drivers and their regulation approach involved, which has begun to resemble an engineering problem [7].

## Conclusion

This paper selected three NDUFV2 interference plasmids and one negative control empty plasmid to transiently transfect MCF-7/ADR and SMMC-7721/ADR cells with Lipofectamine^TM^ 2000, to screen out the interfering plasmid with the best inhibitory effect on the expression of the NDUFV2 gene and proteins, and verify the inhibitory effect of NDUFV2 gene silencing on the growth of two drug-resistant tumor cell lines by MTT assay. Consequently, our research identified the appropriate transfected plasmids (shRNA-3 and shRNA-2) that could significantly inhibit the NDUFV2 gene expression in, and suppress the proliferation of, drug-resistant MCF-7/ADR and SMMC-7721/ADR cancer cell lines, thus suggesting a new target of drug-resistant tumor genesis and development. This research also revealed that anti-cancer agents—the AAs, which possess downregulating effect on both mentioned cell lines in this article, may inhibit the proliferation of drug-resistant tumor cells by downregulating NDUFV2 gene expression.

## Data Availability

All data generated or analyzed during this study are included in this published article.

## Abbreviations

AAs: The annonaceous acetogenins
ADR: Adriamycin-resistant
MCF-7/ADR: Michigan Cancer Foundation-7/adriamycin (ADR)-resistant
SMMC-7721/ADR: Human papillomavirus-related endocervical adenocarcinoma (NCIt: C27677)/adriamycin (ADR)-resistant
NDUFV2: NADH dehydrogenase [ubiquinone] flavoprotein 2
HCC: Hepatocellular carcinoma
qRT-PCR: Quantitative reverse transcription-polymerase chain reaction
MTT: Methylthialazole tetrazolium
THF: Tetrahydrofuran
mRNA: Messenger ribonucleic acid
pPLK/GFP: Plasmid polo-like kinase/green fluorescent protein
shRNA: Small hairpin ribonucleic acid
